# Anti-Wiretap Spectrum-Sharing for Cooperative Cognitive Radio Communication Systems

**DOI:** 10.3390/s19194142

**Published:** 2019-09-24

**Authors:** Peiyuan Si, Weidang Lu, Kecai Gu, Xin Liu, Bo Li, Hong Peng, Yi Gong

**Affiliations:** 1 College of Information Engineering, Zhejiang University of Technology, Hangzhou 310014, China; a18158504979@163.com (P.S.); gukecai2015@163.com (K.G.); ph@zjut.edu.cn (H.P.); 2 School of Information and Communication Engineering, Dalian University of Technology, Dalian 116024, China; liuxinstar1984@dlut.edu.cn; 3 The 54th Research Institute of CECT, Shijiazhuang 050081, China; 4 School of Information and Electrical Engineering, Harbin Institute of Technology, Weihai 264209, China; libo1983@hit.edu.cn; 5 Shenzhen Engineering Laboratory of Intelligent Information Processing for IoT, Southern University of Science and Technology, Shenzhen 518055, China; gongy@sustech.edu.cn

**Keywords:** cognitive radio, cooperative relaying, anti-wiretap, joint resource allocation

## Abstract

As wireless communication technology keeps progressing, people’s requirements for wireless communication quality are getting higher and higher. Wireless communication brings convenience, but also causes some problems. On the one hand, the traditional static and fixed spectrum allocation strategy leads to high wastefulness of spectrum resources. The direction of improving the utility of spectrum resources by combining the advantages of cooperative communication and cognitive radio has attracted the attention of many scholars. On the other hand, security of communication is becoming an important issue because of the broadcasting nature and openness of wireless communication. Physical-layer security has been brought into focus due to the possibility of improving the security in wireless communication. In this paper, we propose an anti-wiretap spectrum-sharing scheme for cooperative cognitive radio communication systems which can secure the information transmission for the two transmission phases of the cooperative communication. We maximized the secondary system transmission rate by jointly optimizing power and bandwidth while ensuring the primary system achieves its secrecy transmission rate. Useful insights of the proposed anti-wiretap spectrum-sharing scheme are given in the simulation results. Moreover, several system parameters are shown to have a big impact for the simulation results.

## 1. Introduction

Recently, due to the progress in wireless communication technology, the number of users supported by the wireless communication system has been increasing, and people’s requirements for transmission rate are getting higher and higher [1,2,3,4]. Wireless communication brings convenience, but also causes some problems such as spectrum scarcity and security problems.

Radio spectrum is a rare and non-renewable precious resource and the demand for radio spectrum resource is expanding rapidly [5,6]. The strategy of radio spectrum allocation is static and fixed, in which relevant the government department divides the spectrum into several frequency bands and reasonably allocates the corresponding frequency bands to the primary users (PU) according to the demand. Even if PU does not use the licensed bandwidth, the other users are still not allowed to use this bandwidth, which results in the waste of wireless spectrum resources.

Cognitive radio is regarded as a prominent solution to solve the problem of spectrum scarcity, which can improve the spectrum use [7,8,9,10]. It provides a flexible and low-cost alternative to wireless devices using classical single-protocol and single-frequency bands. Devices can decrease spectrum wastefulness and fill voids in the wireless spectrum with environment-sensing and environment-adapting. Ref. [11] characterizes the radio frequency spectrum opportunities available in a common global system for mobile (GSM) communications channel to support the operation of a cognitive radio network. Dynamic spectrum access technology gives spectrum managers more available spectrum while secondary users (SU) can share the spectrum dynamically [12,13,14,15]. Spectrum trading is also used to improve spectrum use in different dimensions, e.g., frequency band and time slot, which allows primary users to share its spectrum resource with SU in exchange for a monetary cost [16,17,18]. A new primary system spectrum pricing mechanism is proposed in [17], which takes the preferences of heterogeneous secondary users and various quality in leased spectrum due to diverse interference levels and channel characteristics into account. In [18], researchers considered a cognitive dynamic network architecture in which PU get rewarded if they share their connectivity with SU and act as access points.

Due to the broadcasting character and openness of wireless communication, security has become a serious problem. Broadcast features make the transmission of wireless signals less cryptic, which can lead to information leakage. Signals can be received and carried out as long as the eavesdropper has relevant equipment within a certain distance, which results in communication security risks [19,20,21]. Security attacks include two types: passive attack and active attack [22,23]. Learning or making use of the information of legitimate users are what passive attackers usually do—they do not attack the information itself, i.e., eavesdropping and traffic analysis [24,25]. Active attackers are not only able to involve the process of data modification itself but also interrupt legitimate communication, i.e., DDoS attack [26,27].

There are two main categories of strategy for defending security attacks: new designed networking protocol-based cryptographic encryption approaches, and physical-layer security (PLS) approaches. One of the encryption methods, secured hash function, which can be implemented with several different algorithms, is applied in many fields such as data transfer safety, message authentication, and other user-linked information transfer [28,29]. However, this method is always realized in upper layers, which is challenging to implement in cooperative cognitive radio communication systems. By exploiting the properties of the wireless channel, physical-layer security of relay networks has been remarkable, which is considered to be a quite promising method to improve the security performance of the next-generation wireless communication networks [30,31,32,33]. Ref. [34] proposed a multiple relay-based secure transmission scheme in cognitive radio (CR) communication system. Ref. [35] considered physical-layer security under the scenario where a message transmitted from a secondary source to a secondary destination and the eavesdroppers are poisson spatially distributed. Ref. [36] studied physical-layer security performance based on cooperative two-way cognitive relay with a single passive eavesdropper.

In the existing spectrum-sharing protocol for cooperative cognitive radio communication system, the eavesdropper stops eavesdropping information in the second transmission phase, as it finds that the primary users stop transmitting their signal. However, if the eavesdropper is smart enough, it will find that the primary signal is relayed by the cognitive user in the second transmission phase. Then the eavesdropper will also eavesdrop the primary signal in the second transmission phase. Thus, in this paper, we propose an anti-wiretap spectrum-sharing protocol to secure the information transmission for both transmission phases in a cooperative cognitive radio communication system. Specifically, the transmissions are performed through the following two phases. In the first phase, the primary user transmits the redesigned signal combined by the artificial noise and primary information to jam the eavesdropper. In the second phase, secondary and primary user transmit the primary signal with the designed weight coefficients by using a part of the bandwidth to avoid the eavesdropper eavesdropping the primary information. As a reward, the secondary user can make use of the left bandwidth to transmit its own signal.

The primary contributions of this work are summarized as follows:

- First, we propose an anti-wiretap spectrum-sharing protocol, which can secure the information transmission for both transmission phases in cooperative cognitive radio communication systems.
- Secondly, we formulate a scheme by optimizing power and bandwidth jointly to maximize the secondary system transmission rate while ensuring the required primary system secrecy transmission rate.
- Finally, numerical and simulation results are shown to illustrate the performance of the proposed cooperative spectrum-sharing protocol and reveal the important effects of various system variables.

## 2. System Model and Problem Formulation

### 2.1. System Model

**Figure 1:**
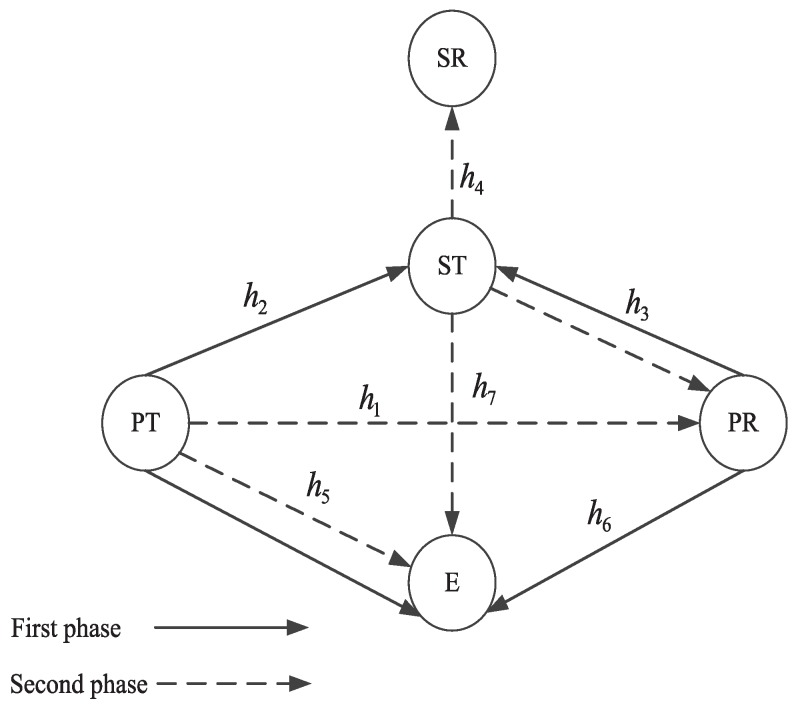
System model.

As shown in Figure 1, the proposed anti-wiretap spectrum-sharing protocol consists of a primary system, a secondary system, and an eavesdropper (E). The primary system contains a primary user (PU), which includes a primary transmitter (PT) and a primary receiver (PR). The secondary system contains a secondary user (SU), which includes a secondary transmitter (ST) and a secondary receiver (SR). When the primary system is in good channel condition, primary information will be sent directly from PT to PR. On the other hand, if the direct link is in a bad channel condition, the secondary system gains the opportunity to access the primary spectrum through forwarding primary information to help it achieve the secrecy transmission rate. We assume that ST is trustworthy, which will not eavesdrop on the primary information when helping PT forward information to PR. We use $h_{i}$, i=1,2,3,…,7, to represent the corresponding channel coefficients. The noise at all nodes is assumed to be complex additive white Gaussian noise (AWGN) with zero mean and unit variance $\sigma^{2}$. The transmit power of PT and ST is denoted as $P_{p}$ and $P_{s}$, respectively.

In the first phase, PT uses all of its bandwidth to transmit the redesigned signal $x_{PT}^{1}$, which is a linearly combined signal of primary signal *s* with power $\beta_{1}P_{p}$ and artificial noise *z* with power $\left(1-\beta_{1}\right)P_{p}$, where $\beta_{1}$ denotes the power allocation coefficient. Then, $x_{PT}^{1}=\sqrt{P_{p}\beta_{1}}s+\sqrt{P_{p}\left(1-\beta_{1}\right)}u_{1}z$. To interfere the eavesdropper, PR also transmits signal $x_{PR}$, which is a signal that contains artificial noise *z* with power $\left(1-\beta_{1}\right)P_{p}$. Then, $x_{PR}=\sqrt{P_{p}(1-\beta_{1}})u_{2}z$, where $u_{1}$ and $u_{2}$ denote the complex weight coefficients. Thus, the received signals at ST and E can be written as

(1) $$\begin{array}{c} r_{ST}=\sqrt{P_{p}\beta_{1}}h_{2}s+\sqrt{P_{p}(1-\beta_{1}})\left(u_{1}h_{2}+u_{2}h_{3}\right)z+n_{ST} \end{array}$$

(2) $$\begin{array}{c} r_{E}^{1}=\sqrt{P_{p}\beta_{1}}h_{5}s+\sqrt{P_{p}\left(1-\beta_{1}\right)}\left(u_{1}h_{5}+u_{2}h_{6}\right)z+n_{E}^{1} \end{array}$$

where $n_{ST}$ and $n_{E}^{1}$ denote the noise received at ST and E in the first phase, respectively.

To guarantee that artificial noise transmitted from PT and PR counteract at ST, $u_{1}$ and $u_{2}$ should satisfy the following conditions:

(3) $$\begin{array}{c} \left\{\begin{array}{c} u_{1}h_{2}+u_{2}h_{3}=0\\ u_{1}^{2}+u_{2}^{2}=1 \end{array}\right. \end{array}$$

Thus, Equation (1) can be rewritten as

(4) $$\begin{array}{c} r_{ST}=\sqrt{P_{p}\beta_{1}}h_{2}s+n_{ST} \end{array}$$

Therefore, the information rate at ST and the eavesdropping rate at E can be written as:

(5) $$\begin{array}{ccc} R_{P}^{1} & = & \frac{1}{2}w\log_{2}\left(1+\beta_{1}\alpha_{2}\right) \end{array}$$

(6) $$\begin{array}{ccc} R_{E}^{1} & = & \frac{1}{2}w\log_{2}\left(1+\frac{\beta_{1}\alpha_{5}}{1+\left(1-\beta_{1}\right)\alpha_{m}}\right) \end{array}$$

where $\alpha_{2}=\frac{P_{p}{\left|h_{2}\right|}^{2}}{\sigma^{2}}$, $\alpha_{5}=\frac{P_{p}{\left|h_{5}\right|}^{2}}{\sigma^{2}}$ and $\alpha_{m}=\frac{P_{p}{\left|u_{1}h_{5}+u_{2}h_{6}\right|}^{2}}{\sigma^{2}}$.

In the second phase, ST uses a part of the licensed spectrum bw authorized by the primary system and power $\beta_{2}P_{s}$ to forward the received primary information to PR with decode-and-forward relaying protocol, by transmitting the signal $x_{ST}=\sqrt{P_{s}\beta_{2}}v_{1}s$. To prevent E eavesdropping on the primary information, PT also transmits the signal $x_{PT}^{2}=\sqrt{P_{p}\beta_{2}}v_{2}s$, where $v_{1}$ and $v_{2}$ denote the complex weight coefficients.

Thus, the received signal at PR and E can be written as

(7) $$\begin{array}{ccc} r_{PR} & = & \sqrt{\beta_{2}}\left(\sqrt{P_{s}}v_{1}h_{3}+\sqrt{P_{p}}v_{2}h_{1}\right)s+n_{PR} \end{array}$$

(8) $$\begin{array}{ccc} r_{E}^{2} & = & \sqrt{\beta_{2}}\left(\sqrt{P_{s}}v_{1}h_{7}+\sqrt{P_{p}}v_{2}h_{5}\right)s+n_{E}^{2} \end{array}$$

where $n_{PR}$ and $n_{E}^{2}$ denotes the noise received at PR and E in the second phase, respectively.

To prevent E eavesdropping on the primary information in the second phase, $v_{1}$ and $v_{2}$ should satisfy the following conditions:

(9) $$\begin{array}{c} \left\{\begin{array}{c} \sqrt{P_{s}}v_{1}h_{7}+\sqrt{P_{p}}v_{2}h_{5}=0\\ v_{1}^{2}+v_{2}^{2}=1 \end{array}\right. \end{array}$$

Therefore, the eavesdropping rate at E in the second phase is zero and the information rate at PR can be written as:

(10) $$R_{P}^{2}=\frac{1}{2}bw\log_{2}\left(1+\beta_{2}\alpha_{n}\right)$$

where $\alpha_{n}=\frac{P_{s}|v_{1}h_{3}{|}^{2}+P_{p}{\left|v_{2}h_{1}\right|}^{2}}{\sigma^{2}}$.

Thus, the information rate of primary system and eavesdropping rate at E through two phases transmission can be written as:

(11) $$\begin{array}{ccc} R_{P} & = & \min\{R_{P}^{1},R_{P}^{2}\} \end{array}$$

(12) $$\begin{array}{ccc} R_{E} & = & R_{E}^{1} \end{array}$$

Then, the secrecy transmission rate of primary system can be written as

(13) $$R_{SEC}=R_{P}-R_{E}$$

As a reward for forwarding the primary signal, ST will be permitted to use the remained spectrum and power to transmit its own signal *x* to SR in the second phase. Then, the received signal at SR can be written as

(14) $$r_{SR}=\sqrt{P_{s}\left(1-\beta_{2}\right)}h_{4}x+n_{SR}$$

Thus, the information rate of secondary system can be written as:

(15) $$R_{S}=\frac{1}{2}\left(1-b\right)w\log_{2}\left(1+\left(1-\beta_{2}\right)\alpha_{4}\right)$$

### 2.2. Problem Formulation

With the objective of maximizing the information rate of secondary system with the primary secrecy transmission rate constraint, through joint optimizing the power allocation $\beta_{1},\beta_{2}$ and bandwidth allocation *b*, the optimization problem can be formulated as

(16) $$\max_{\beta_{1},\beta_{2},b}\;R_{S}$$

subject to

(17) $$\begin{array}{c} \left\{\begin{array}{c} R_{SEC}\geq R_{T}\\ 0\leq \beta_{1}\leq 1\\ 0\leq \beta_{2}\leq 1\\ 0\leq b\leq 1 \end{array}\right. \end{array}$$

where $R_{T}$ represents the target secrecy transmission rate of the primary system.

## 3. Optimal Solutions

In this section, we will optimize bandwidth and power allocation jointly under the primary system target secrecy rate constraint.

For convenience of expression, we define

(18) $$\begin{array}{c} \left\{\begin{array}{c} R_{2}=w\log_{2}\left(1+\alpha_{2}\beta_{1}\right)\\ R_{3}=w\log_{2}\left(1+\alpha_{n}\beta_{2}\right)\\ R_{4}=w\log_{2}\left(1+\left(1-\beta_{2}\right)\alpha_{4}\right) \end{array}\right. \end{array}$$

Thus, the optimization problem in Equation (16) can be written as

(19) $$\max_{\beta_{1},\beta_{2},b}\;\frac{1}{2}\left(1-b\right)R_{4}$$

subject to

(20) $$\begin{array}{c} \left\{\begin{array}{c} \frac{1}{2}R_{2}-R_{E}\geq R_{T}\\ \frac{1}{2}bR_{3}-R_{E}\geq R_{T}\\ 0\leq \beta_{1}\leq 1\\ 0\leq \beta_{2}\leq 1\\ 0\leq b\leq 1 \end{array}\right. \end{array}$$

Due to the non-convex constraints in Equation (20), it is difficult to obtain the optimal solution directly. We solve the above optimization problem through the following three steps. We will show in the numerical results that the above solution achieves the optimal performance which can be proved through the exhaustive search scheme.

### 3.1. Finding Optimal Bandwidth Allocation $b^{*}$ with Fixed Power Allocation $\beta_{1}$ and $\beta_{2}$

To satisfy the second condition of Equation (20), we can obtain

(21) $$b\geq \frac{2(R_{T}+R_{E})}{R_{3}}$$

From Equation (15), we can find that the target function $R_{S}$ is a monotonic decreasing function of *b* with fixed $\beta_{1}$ and $\beta_{2}$. Therefore, we can find the optimal bandwidth allocation $b^{*}$ as

(22) $$b^{*}=\frac{2(R_{T}+R_{E})}{R_{3}}=\frac{2\left(R_{T}+\frac{1}{2}w\log_{2}\left(1+\frac{\beta_{1}\alpha_{5}}{1+\left(1-\beta_{1}\right)\alpha_{m}}\right)\right)}{w\log_{2}\left(1+\alpha_{n}\beta_{2}\right)}$$

### 3.2. Finding Optimal Power Allocation $\beta_{1}^{*}$ with Fixed $\beta_{2}$

Substituting the optimal bandwidth allocation $b^{*}$ into $R_{S}$, we can obtain

(23) $$R_{S}=\frac{1}{2}\left(1-\frac{2\left(R_{T}+\frac{1}{2}w\log_{2}\left(1+\frac{\beta_{1}\alpha_{5}}{1+\left(1-\beta_{1}\right)\alpha_{m}}\right)\right)}{w\log_{2}\left(1+\alpha_{n}\beta_{2}\right)}\right)w\log_{2}\left(1+\left(1-\beta_{2}\right)\alpha_{4}\right)$$

To satisfy the first condition of Equation (20), we can obtain

(24) $$\frac{1}{2}w\log_{2}\left(1+\beta_{1}\alpha_{2}\right)-\frac{1}{2}w\log_{2}\left(1+\frac{\beta_{1}\alpha_{5}}{1+\left(1-\beta_{1}\right)\alpha_{m}}\right)\geq R_{T}$$

After some manipulation, Equation (24) can be rewritten as

(25) $$\begin{array}{c} f\left(\beta_{1}\right)=A\beta_{1}^{2}+B\beta_{1}+C\geq 0 \end{array}$$

where $A=-\alpha_{2}\alpha_{m}$, $B=\alpha_{2}\left(1+\alpha_{m}\right)-\alpha_{m}-2^{\frac{2R_{T}}{w}}\left(\alpha_{5}-\alpha_{m}\right)$ and $C=\left(1+\alpha_{m}\right)\left(1-2^{\frac{2R_{T}}{w}}\right)$.

Assuming $x_{1}=\frac{-B+\sqrt{B^{2}-4AC}}{2A}$ and $x_{2}=\frac{-B-\sqrt{B^{2}-4AC}}{2A}$ are the two roots of the equation $f(\beta_{1})=0$. It is easy to find that A<0 and C<0. Thus, if $-\frac{B}{2A}<0$, we can find that two roots $x_{1}$ and $x_{2}$ are negative. Then, there will be no positive value of $\beta_{1}$ that can satisfy the condition in Equation (25). Thus, we can conclude that $-\frac{B}{2A}\geq 0$. Due to C<0, we can obtain $0<x_{1}<x_{2}$. From Equation (23), we can find that $R_{S}$ is a monotonic decreasing function of $\beta_{1}$ with fixed $\beta_{2}$, thus the optimal value of $\beta_{1}^{*}$ depends on whether $x_{1}$ is larger than 1. If $x_{1}$ is smaller than 1, $\beta_{1}^{*}=x_{1}$, otherwise there will be no optimal value of $\beta_{1}^{*}$ to satisfy the condition $0\leq \beta_{1}\leq 1$.

### 3.3. Finding Optimal Power Allocation $\beta_{2}^{*}$

Substituting the optimal power allocation $\beta_{1}^{*}$ into $R_{s}$, we can obtain

(26) $$\begin{array}{c} R_{s}=\frac{1}{2}w\log_{2}\left(1+\left(1-\beta_{2}\right)\alpha_{4}\right)-\left(R_{T}+R_{E}\right)\frac{w\log_{2}\left(1+\left(1-\beta_{2}\right)\alpha_{4}\right)}{w\log_{2}\left(1+\beta_{2}\alpha_{n}\right)} \end{array}$$

To satisfy the fifth condition of Equation (20), we can obtain $\beta_{N}\leq \beta_{2}\leq 1$, where $\beta_{N}=\frac{2^{\frac{2(R_{T}+R_{E})}{w}}-1}{\alpha_{n}}$.

From Equation (26), it is easy to find that $R_{S}$ is composed of two decreasing function of $\beta_{2}$. Let $\beta =1-\beta_{2}$, then $R_{S}$ is composed of two increasing function of β. After some manipulation, we can obtain

(27) $$\begin{array}{ccc} R_{S}\left(\beta \right) & = & f(\beta )-g(\beta ) \end{array}$$

where $f\left(\beta \right)=\frac{1}{2}w\log_{2}\left(1+\beta \alpha_{4}\right)$ and $g\left(\beta \right)=\left(R_{T}+R_{E}\right)\frac{w\log_{2}\left(1+\beta \alpha_{4}\right)}{w\log_{2}\left(1+\left(1-\beta \right)\alpha_{n}\right)}$.

Introducing a new variable *t*, $g\left(\beta \right)+t=g{\left(\beta \right)}_{max}$, which satisfies $0\leq t\leq \left(g{\left(\beta \right)}_{max}-g{\left(\beta \right)}_{min}\right)$, $R_{S}$ can be rewritten as

(28) $$R_{S}\left(\beta ,t\right)=f\left(\beta \right)+t-g{\left(\beta \right)}_{max}$$

From Equation (28), we can find that it is a monotonic optimization problem which can be solved by the polyblock outer approximation approach [37,38], which is formed by constructing Polyblock covering feasible region D step by step. Feasible region D is composed of the intersection of a NormalSet and a ReverseNormalSet. Polyblock outer approximation approach is realized as follows: First, choose a block [l,u] as original Polyblock. Let $z^{k}$ denote the vertex which makes the objective function achieve the maximum value among all the vertex in the *k*th iteration. Let $x^{k}$ denote the intersection of the line between *l* and $z^{k}$ and the feasible region D in the *k*th iteration. The Polyblock is gradually specified by splitting $\left[x^{k},z^{k}\right]$ from block $\left[l,z^{k}\right]$ in each iteration. Through alternating *n* components, let one component be equal to the component of $x^{k}$, and the other components be equal to the components of $z^{k}$, which will result in *n* new vertices. The iteration stops when the difference between the upper bound (the maximum target value of the vertex) and lower bound (the target value of the current best boundary point) achieves at a given precision.

Thus, our optimization problem can be solved by the polyblock outer approximation approach as shown in Algorithm 1.

**Algorithm 1** Polyblock Outer Approximation Algorithm
Step one:Initialization    1.Choose the lower angular point $\rho_{min}$ and the upper angular point $\rho_{max}$ of Polyblock. Initializes the current optimal target value CBV=∞, current optimal value CBS=∅. Set iteration index k=1 and error tolerance ϵ≪1.    2.Initialize the vertex set $\Gamma_{1}=\left\{0\right\}$, set algorithm terminating mark $f_{stop}=0$. For convenience of expression, define $R_{S}\left({\left\{\rho_{i}\right\}}_{i\in \Gamma_{1}}\right)=\frac{1}{2}w\log_{2}\left(1+\beta \alpha_{4}\right)+t-g{\left(\beta \right)}_{max}$.Step two:Iteration    3.Traverse vertex set $\Gamma_{1}$, select the vertex that belongs to CBV and update $\Gamma_{1}$.    4.Judge whether $\Gamma_{1}$ is empty. If $\Gamma_{1}$ is empty, set $f_{stop}=1$, go to 14. Else, go to 5.    5.Chose a vertex $z^{k}$ that maximize the objective function from set $\Gamma_{1}$, expressed as $z^{k}\in arg\;max\left\{V\left({\left\{\rho_{i}\right\}}_{i\in \Gamma }\right)|{\left\{\rho_{i}\right\}}_{i\in \Gamma }\in \Gamma_{k}\right\}$.    6.Judge whether $z^{k}$ is repeated with the former optimal vertex. If the number of consecutive repetitions is larger than a given value, set $f_{stop}=1$, go to 14. Else, go to 7.    7.Construct a straight line connecting $z^{k}$ and $\rho_{min}$    8.Find the intersection point of the line constructed in 7 and the upper boundary of the feasible region using dichotomy.    9.If $V(x^{k})<CBV$, go to 10, else go to 11.   10.Update $CBV=V(x^{k})$, set $CBS=x^{k}$.   11.If $\left|\right|x^{k}-z^{k}\left|\right|<\epsilon$, set $f_{stop}=1$, go to 14. Else, go to 12.   12.Update the current vertex set $\Gamma_{k+1}=\left(\Gamma_{k}\setminus \left\{z^{k}\right\}\right)\cup \left\{z^{k}+\left(x_{i}^{k}-z_{i}^{k}\right)e_{i},\forall i\in \Gamma \right\}$ and delete the vertexes not belong to $G_{\left(\rho_{0}\right)}$.   13.If $\Gamma_{k+1}$ is empty, set f(stop)=1, go to 14. Else k=k+1, return to 4.Step three:Output   14.Output $CBS={\left\{\rho_{i,(\rho_{0})}^{*,sub}\right\}}_{i\in \Gamma_{1}}$, $R_{s}\left(\rho_{0}\right)=CBV$.


## 4. Simulation Results and Discussion

**Figure 2:**
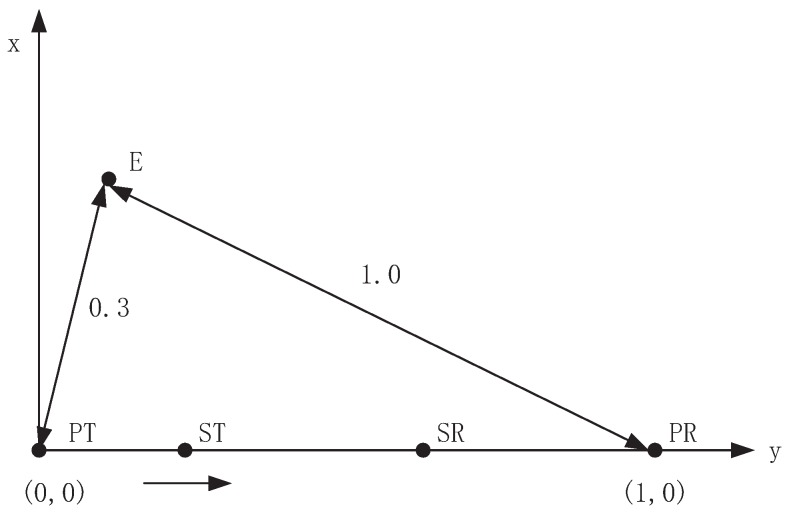
Position of PT, PR, ST, SR and E.

In this section, we investigate the performance of proposed anti-wiretap spectrum-sharing strategy. As shown in Figure 2, PT, PR, ST, SR and E are distributed in a two-dimensional X−Y plane, in which PT is located at (0,0), PR is located at (1,0) and ST moves from point (0,0) to (1,0). The distance of ST to SR is half of the distance of ST to PR. Thus, we can obtain $d_{1}=1,d_{2}=1-d_{3},d_{4}=d_{3}/2$. The distance of E to PT is $d_{5}=0.3$, the distance of E to PR is $d_{6}=1$ and the distance of E to ST is $d_{7}=d_{4}$. Set the path loss coefficient to be v=−3 and bandwidth is w=1.

**Figure 3:**
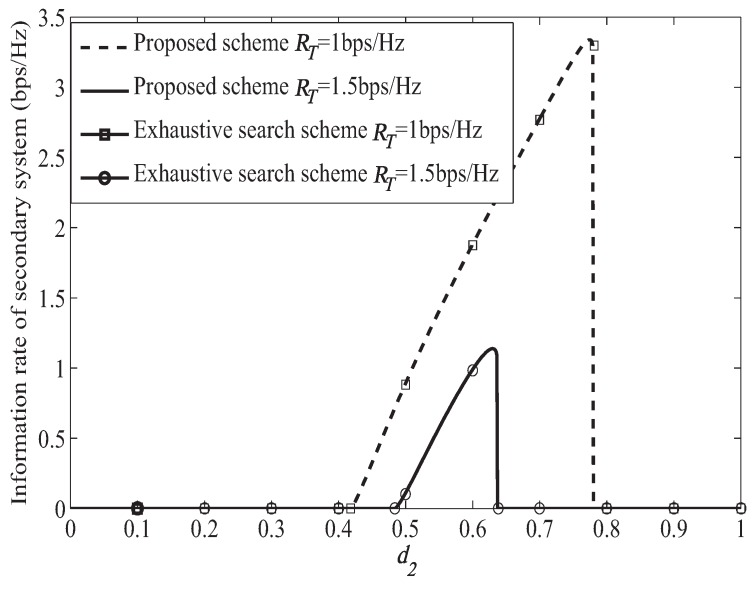
Transmission rate of cognitive user under different target secrecy rate.

**Figure 4:**
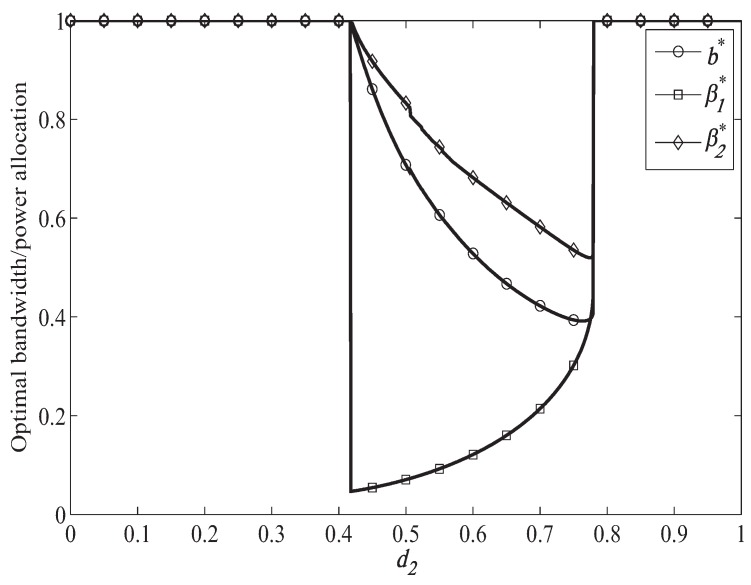
Optimal resource allocation when $R_{T}$ = 1.0 bps/Hz.

**Figure 5:**
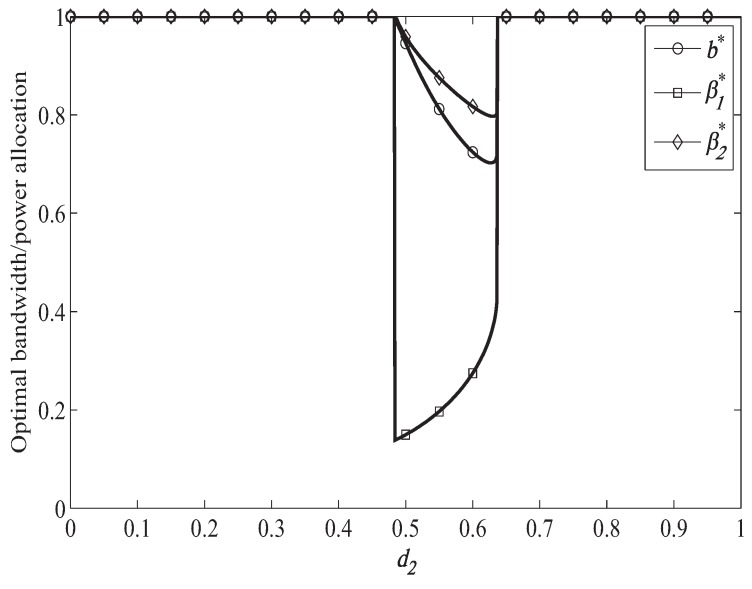
Optimal resource allocation when $R_{T}$ = 1.5 bps/Hz.

Figure 3 shows the information rate of the secondary system when ST moves from (0,0) to (1,0) under different primary system target secrecy rate. In Figure 3, we can find that the performance of our proposed scheme and exhaustive search scheme are the same, which verifies the effectiveness our proposed scheme. In exhaustive search scheme, the optimal power and bandwidth allocation is obtained with the bisection method. In Figure 3, we can find that the information rate of secondary system becomes smaller when the primary system target secrecy rate becomes larger. It is because that more bandwidth and power will be allocated to forward the primary signal when helping the primary system achieve larger target secrecy rate, which can be illustrated from Figure 4 and Figure 5. Then, less bandwidth and power are left for transmitting the secondary signal, which leads smaller information rate of the secondary system. When $R_{T}=1.0$ bps/Hz, the secondary system can access to the primary spectrum only when $0.42<d_{2}<0.78$. The secondary system cannot access to the primary spectrum when $d_{2}\leq 0.42$, which is because that in this case the distance of ST to PR is too far away, leading poor channel condition for the secondary system to help forward the primary signal to PR. Then, the primary system cannot achieve its target secrecy rate. Thus, the secondary system will not be permitted to access to the primary spectrum. When $d_{2}\geq 0.78$, the channel condition between PT and ST is too poor for the secondary system to help the primary system achieve the target secrecy rate. Thus, the secondary system cannot access to the primary spectrum. When $R_{T}=1.5$ bps/Hz, the similar case happened when ST located in $0.48<d_{2}<0.64$. In the access range, the information rate of the secondary system becomes larger when $d_{2}$ becomes larger. It is because that when $d_{2}$ becomes larger, which means that ST gets closer to PR. Then the channel condition between ST and PR becomes better, which will lead to less bandwidth and power to forward the primary signal as illustrated in Figure 4 and Figure 5. Thus, more bandwidth and power can be used to transmit the secondary signal leading larger information rate of the secondary system.

Figure 4 shows the optimal bandwidth and power allocation when ST moves from (0,0) to (1,0), when the primary target secrecy rate is $R_{T}=1$ bps/Hz. In Figure 4, we can find that in the access range the power and bandwidth allocated to help forward becomes smaller when $d_{2}$ becomes larger. It is because that the channel condition between ST and PR becomes better when $d_{2}$ becomes larger, which means that the secondary system can reduce bandwidth and power to help the primary system achieve the target secrecy rate. We can also observe form Figure 4 that when $d_{2}$ becomes larger, the power used to transmit the artificial noise will becomes smaller. It is because that the information rate of the secondary system becomes larger when $d_{2}$ becomes larger, which leads less power to transmit the artificial noise to interfere the eavesdropper.

Figure 5 shows the optimal bandwidth and power allocation when ST moves from (0,0) to (1, 0), when the primary target secrecy rate is $R_{T}=1.5$ bps/Hz. Compared to Figure 4, we can find that the access range of the secondary system becomes smaller when the primary system target secrecy rate becomes larger, which is because that better channel is needed to forward the primary signal when helping the primary system achieve larger target secrecy rate. In Figure 5, we can also observe that more bandwidth and power will be allocated to forward the primary signal when helping the primary system achieve larger target secrecy rate with a fixed $d_{2}$.

## 5. Conclusions

In this paper, we proposed an anti-wiretap spectrum-sharing scheme for cooperative cognitive radio communication systems which can secure the information transmission for the two transmission phases of the cooperative communication. To secure the information transmission in phase 1, PT transmits the redesigned signal which is combined by the artificial noise and primary information to jam the eavesdropper. To secure the information transmission in the phase 2, PT and ST transmit the primary information with the designed weight coefficients by using a part of the bandwidth and power to avoid the eavesdropper eavesdropping the primary information. As a reward, the secondary user can used the remaining bandwidth to transmit its own information. The joint optimization of bandwidth and power allocation is formulated to maximize the secondary system information rate while ensuring the primary system achieve its secrecy transmission rate. In simulation results, we give some useful insights of the proposed anti-wiretap spectrum-sharing scheme and reveal the system parameter impact for the system performance.
